# Jarid1b promotes epidermal differentiation by mediating the repression of Ship1 and activation of the AKT/Ovol1 pathway

**DOI:** 10.1111/cpr.12638

**Published:** 2019-05-31

**Authors:** Xuewei Sun, Zhiyuan Li, Yanfang Niu, Lijuan Zhao, Yichuan Huang, Qiang Li, Shengnan Zhang, Ting Chen, Tao Fu, Tao Yang, Xiaofei An, Yan Jiang, Jisheng Zhang

**Affiliations:** ^1^ Department of Otolaryngology‐Head and Neck Surgery, Key Laboratory, The Affiliated Hospital of Qingdao University Qingdao University Qingdao China; ^2^ Department of Andrology, The Affiliated Hospital of Qingdao University Qingdao University Qingdao China; ^3^ Department of Biochemistry & Molecular Biology Shanxi Medical University Taiyuan China; ^4^ Department of Endocrinology, Jiangsu Province Hospital of Chinese Medicine Affiliated Hospital of Nanjing University of Chinese Medicine Nanjing China; ^5^ Shandong Key Laboratory of Digital Medicine and Computer Assisted Surgery Qingdao China; ^6^ Shandong College Collaborative Innovation Center of Digital Medicine in Clinical Treatment and Nutrition Health Qingdao China

**Keywords:** differentiation, epidermis, Jarid1b, Ovol1, Ship1

## Abstract

**Objectives:**

Terminally differentiated stratified squamous epithelial cells play an important role in barrier protection of the skin. The integrity of epidermal cells is maintained by tight regulation of proliferation and differentiation. The aim of this study was to investigate the role of epigenetic regulator H3K4me3 and its demethylase Jarid1b in the control of epithelial cell differentiation.

**Materials and methods:**

RT‐qPCR, Western blotting and IHC were used to detect mRNA and protein levels. We analysed cell proliferation by CCK8 assay and cell migration by wound healing assay. ChIP was used to measure H3K4me3 enrichment. A chamber graft model was established for epidermal development.

**Results:**

Our studies showed that H3K4me3 was decreased during epidermal differentiation. The H3K4me3 demethylase Jarid1b positively controlled epidermal cell differentiation in vitro and in vivo. Mechanistically, we found that Jarid1b substantially increased the expression of mesenchymal‐epithelial transition (MET)‐related genes, among which Ovol1 positively regulated differentiation gene expression. In addition, Ovol1 expression was repressed by PI3K‐AKT pathway inhibitors and overexpression (O/E) of the PI3K‐AKT pathway suppressor Ship1. Knockdown (KD) of Ship1 activated downstream PI3K‐AKT pathway and enhanced Ovol1 expression in HaCaT. Importantly, we found that Jarid1b negatively regulated Ship1 expression, but not that of Pten, by directly binding to its promoter to modulate H3K4me3 enrichment.

**Conclusion:**

Our results identify an essential role of Jarid1b in the regulation of the Ship1/AKT/Ovol1 pathway to promote epithelial cell differentiation.

## 1 INTRODUCTION

The integrity of stratified epithelia is maintained by tight regulation of proliferation and differentiation. Basal cell self‐renewal maintains the cell population and gives rise to differentiated cells, which maintains tissue homeostasis and the skin barrier function to defend against environmental stresses and protect the body from dehydration.^1^ Aberrant keratinocyte differentiation is involved in many skin diseases, such as psoriasis, atopic dermatitis and skin cancers. Increasing evidence has documented that the switch to differentiation gene expression is epigenetically controlled and that histone modifications are involved in the cell fate commitment. Repressive H3K27me3 is enriched in non‐epidermal and differentiation genes to sustain the stem state. Knocking out key subunits of the PRC2 complex, Ezh1/2, thereby inducing the ablation of H3K27me3 marks, leads to premature differentiation of keratinocytes and Merkel cells.^2^, ^3^, ^4^ H3K4me3 is usually found around transcription start sites as an active gene transcription marker. The transient poised state of H3K4me3 and H3K27me3 is resolved upon differentiation, with only one of the marks remaining at the promoter to stably activate or silence gene expression, which has been proposed to occur in embryonic stem cells (ESCs), induced pluripotent stem cells, some non‐stem cell lines and cancer cells.^5^ Intriguingly, global loss of H3K4me3 accompanies the differentiation of ESCs to progenitor cells.^6^

Lysine demethylase Jarid1 family members, including Jarid1a, Jarid1b, Jarid1c and Jarid1d, have been found to demethylase H3K4me3.^7^ The role of Jarid1b in cell differentiation has recently received increased attention. Jarid1b contributes to the myogenic, osteogenic and macrophage lineage specification of mesenchymal cells.^8^, ^9^, ^10^ Silencing of Jarid1b in ESCs impairs neural linage commitment.^11^ An RNAi screen identified Jarid1b as a major regulator of haematopoietic stem cell differentiation.^12^ Jarid1b contributes to trophoblast stem cell differentiation by downregulating self‐renewal genes via removing H3K4 methylation.^13^ Jarid1b has been reported to be highly expressed in various types of tumours. In luminal breast cancer, Jarid1b is a luminal lineage‐driving oncogene.^14^ Leukaemia stem cell (LSC) differentiation is associated with downregulation of H3K4me3 profiles, and overexpression (O/E) of Jarid1b leads to LSC differentiation, reducing leukaemia stem cell oncogenic potential.^15^ While these studies provide insight into the role of H3K4me3 in cell fate decisions and that of Jarid1b in regulating the differentiation of a variety of cell types, it is unclear how Jarid1b contributes to stratified epithelium differentiation.

Therefore, to investigate the role of H3K4me3 demethylases in regulating stratified epithelium differentiation, we evaluated the global expression levels of H3K4me3 and its demethylases in the Jarid1 family. Our findings demonstrated that at the global level, H3K4me3 expression decreased upon keratinocyte differentiation and that both methyltransferases and demethylases of H3K4me3 were increased, suggesting a crucial role of demethylases during differentiation. Furthermore, overexpression of Jarid1b promoted keratinocyte differentiation, whereas depletion of Jarid1b delayed differentiation in vitro and impaired differentiation layer formation in reconstituted epidermis in vivo. Mechanistically, we demonstrate that Jarid1b regulates the key differentiation transcription factor Ovol1 by directly binding to the PI3K‐AKT pathway suppressor Ship1, but not to Pten. These findings suggest an epigenetic role of Jarid1b in regulating keratinocyte differentiation.

## 2 MATERIALS AND METHODS

### 2.1 Animals

Nude mice (male, 6 weeks old, 20‐25 g) were purchased from the Beijing Vital River Laboratory, China, and housed in the SPF‐grade centre of the animal facility in the affiliated hospital of Qingdao University. All experimental protocols were approved by the ethics committee on animal care of the Affiliated Hospital of Qingdao University. All animal studies were performed in strict accordance with the approved protocol.

### 2.2 Cell culture and treatment with inhibitors

HaCaT cells were cultured in low‐calcium growth medium. Briefly, calcium‐restricted media were obtained from Gibco, and calcium in serum was removed by incubation with Chelex 100 (Bio‐Rad 142‐2842).^16^ Low‐calcium growth medium and high‐calcium differentiation medium were produced by adding CaCl_2_ at 0.03 mmol/L and 2.8 mmol/L, respectively. Cells were treated with inhibitors (Selleck) at the indicated concentration for 24 hours.

### 2.3 Plasmids and transfection

Jarid1b and Ovol1 were subcloned into the cumate‐inducible Cumate‐pLenti‐GIII‐CMV‐IRES‐puro‐SV40‐GFP vector. In this system, the repressed CymR target gene is expressed when cumate is present. Ship1 was subcloned into the pCDNA3‐3Flag vector. The target shRNA sequence was chemically synthesized in the pLKO.1 vector, which is included in a Tables [Link], [Link], [Link]. Lentivirus vectors were transfected into HEK293FT cells with the helper plasmids delta8.9 and VSVG. The supernatant was collected after 48 hours of transfection and centrifuged at 50 000 *g* at 4°C for 3 hours. The virus pellet was redissolved in an appropriate volume. An inducible cell line was infected with the CymR virus and selected with hygromycin to obtain a pure population. Then, CymR‐expressing cells were infected with cumate‐inducible lentivirus and selected by puromycin. Target gene expression was induced via cumate treatment at the indicated concentration when necessary. The Ubi‐Pten‐3FLAG and control plasmids were purchased from GeneChem Company, China. Transient transfection was performed with Lipofectamine 3000 following the instructions of the manufacturer.

### 2.4 Western blot analysis

Cells were lysed in 2% SDS lysis buffer and sonicated. A total of 15 μg of protein was loaded after quantification (Pierce 23225). Then, the proteins were transferred to a 0.45 μm PVDF membrane. After 1 hour of blocking with 5% BSA, the membrane was incubated with the primary antibody overnight at 4°C and then the secondary antibody at room temperature for 1 hour on the next day. Antibody information can be found in Tables [Link], [Link], [Link]. After washing, the blots were developed with the Super Signal Pico substrate (Pierce Biotechnology).

### 2.5 Real‐time reverse‐transcription PCR

Total RNA was isolated by using RNAiso Plus (Takara D9108) and reverse‐transcribed using All‐In‐One RT MasterMix with the AccuRT Genomic DNA Removal Kit (Abm G492). Quantitative PCR amplification (Abm MasterMix‐S) using a Roche LightCycler 480 was performed via initial denaturation at 95°C for 5 minutes, followed by 40 cycles of 95°C for 10 s and 60°C for 15 s. The sequences of primers can be found in Tables [Link], [Link], [Link]. Relative quantification was performed using the 2^−ΔΔCt^ method normalized to GAPDH.

### 2.6 Chromatin immunoprecipitation (ChIP)

The ChIP procedure has been described previously.^17^, ^18^ In brief, formaldehyde cross‐linked HaCaT cells were sonicated for 200 cycles (25 seconds ON and 15 seconds OFF, 40% amplitude) with the 2 mm probe of VCX750 sonicator. Dynal Protein G magnetic beads (Thermo 10003D) were incubated with an antibody overnight and then with the sonicated samples for at least 16 hours at 4°C. The conjugated beads were washed with low‐salt, high‐salt, LiCl and TE buffers in turn for 45 minutes each. DNA was isolated with phenol/chloroform/isoamyl alcohol after reverse cross‐linking and RNase A and proteinase K treatment.

### 2.7 Immunohistochemistry and immunofluorescence

For immunohistochemistry, antigen retrieval was performed with pH 6.0 citrate buffer for paraffin‐embedded tissue sections. After removing endogenous peroxidase with 0.3% hydrogen peroxide and non‐specific protein blocking reagent incubation, primary antibody incubation was conducted overnight at 4°C. The mean intensity was quantified by using Image‐Pro Plus from at least three raw, single‐channel greyscale images that were obtained under the same conditions.

For immunofluorescence, 4% PFA‐fixed cells were blocked with blocking buffer for 1 hour and incubated with the primary antibody overnight at 4 °C. After washing three times, the cells were incubated with the secondary antibody for 1 hour at room temperature, followed by Hoechst counterstaining.

### 2.8 Wound healing assay

A total of 600 000 cells were seeded in six‐well plate wells. The next day, scratches were introduced using 200 µL pipet tips to create scratches with a similar diameter. The cells were washed once with PBS to remove floating cells. Then, 2 mL of fresh media supplemented with 1% FBS and the indicated concentration of cumate was added. Images of the scratches were recorded at the indicated time points at the same positions. The wound areas were quantified with ImageJ software.

### 2.9 Cell viability

For the CCK8 assay, cells were seeded at density of 1 × 10^4^ per well in a 96‐well plate. Following the instructions of the Cell Counting Kit‐8 (Dojindo CK04), the attached cells were incubated with 110 μL of medium containing 10 μL of CCK‐8 solutions for 30 minutes to 2 hours. The absorbance was measured at 450 nm.

A live‐cell imaging system was used to evaluate cell viability upon Jarid1b knockdown (KD). A total of 5000 cells per well were transfected with control or Jarid1b shRNA under low‐ or high‐calcium culture conditions, and images were obtained every 6 hours. Cell confluence was calculated to reflect cell proliferation.

### 2.10 Preparation of skin cell suspensions and chamber grafting

Engraftments were performed as described previously.^19^, ^20^ Neonatal mice were sacrificed within 24 hours, and the back skin was dissected. The skin was floated on 1 U/mL dispase II overnight at 4°C. The next morning, the epidermis and dermis were separated with sharp forceps. The dermis was digested with 0.1% collagenase type 1 (LS004196) and 0.005% DNase I (LS002058) at 37°C for 1 hour with agitation, and this step was repeated twice. Collagenase activities were quenched by DMEM containing 10% foetal bovine serum. Single dermal cells were filtered through a 40 μm cell strainer. HaCaT cells were trypsinized to obtain a single‐cell suspension. All cell lines were counted. Equal numbers of HaCaT and dermal cells were combined at 2 × 10^4^ cells/μL in a 500 μL volume. The combined cells were injected into a silicon chamber implanted in the back muscle fascia of anaesthetized nude mice (Beijing Vital River Laboratory, China). The chambers were removed after 1 week. The reconstituted skins were collected after 27 days of implantation.

### 2.11 Statistical analysis

To determine the significance of data obtained from human samples or cell culture assays, comparisons were performed using descriptive and inferential statistics accompanied by graphs from the Prism software program (GraphPad). In all column bar graphs, the mean value ± 1 standard deviation is presented. For all the statistics, the 0.05 level of confidence was accepted for statistical significance.

## 3 RESULTS

### 3.1 H3K4me3 expression decreases and Jarid1b expression increases during epidermal differentiation

**Figure 1:**
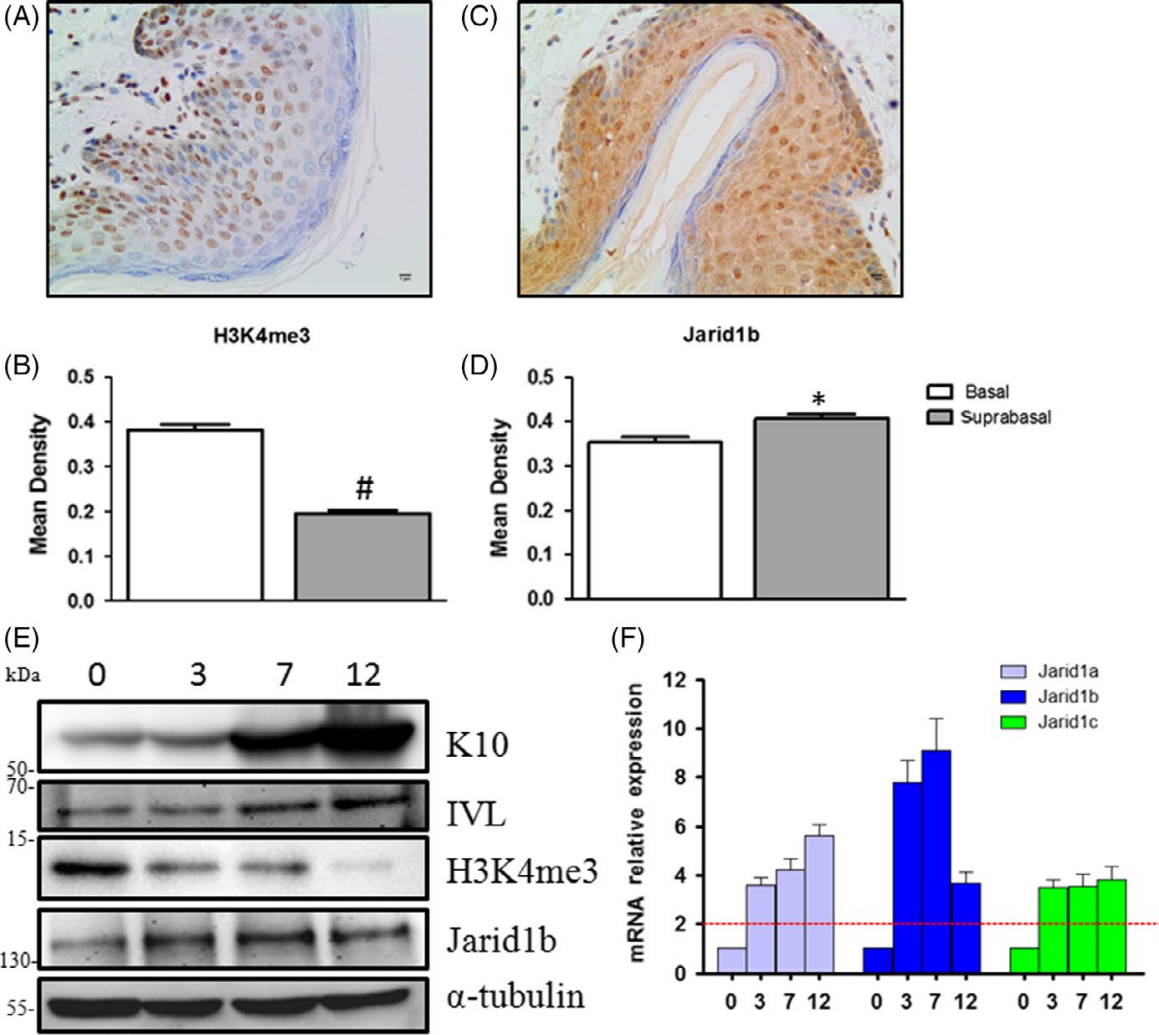
H3K4me3 expression decreased and Jarid1b expression increased during epidermal differentiation. Representative images of immunohistochemical staining for (A) H3K4me3 and (C) Jarid1b in human foreskin. B, H3K4me3 expression was decreased in the suprabasal layers compared with the basal layer (*P* < 0.01), whereas (D) Jarid1b expression was increased (*P* < 0.05). E, Western blot analysis of the expression of keratinocyte differentiation markers (K10 and IVL) and (F) RT‐qPCR analysis of the mRNA expression of Jarid1 family members in HaCaT cells grown in low (day 0)‐ and high‐calcium media for 3, 7 and 12 days

The epidermis offers an ideal in vivo model for investigating cell differentiation due to the physical separation of differentiated suprabasal layers from basal layer cells. Immunohistochemistry assays demonstrated that H3K4me3 expression was decreased (Figure 1A,B) in suprabasal cells compared with basal cells, while Jarid1b expression was increased (Figure 1C,D). Calcium‐induced differentiation has been widely accepted as an in vitro model for investigating epidermal differentiation. HaCaT cells were cultured in low‐calcium conditions to maintain an undifferentiated state and underwent differentiation upon high‐calcium stimulation.^21^ The results showed that the expression of K10 and involucrin (IVL) increased with time in high‐calcium conditions, indicating that differentiation was successfully induced in our system. We found that H3K4me3 decreased upon differentiation (Figure 1E). Next, we examined the gene expression of methyltransferases and demethylases related to H3K4me3. We found that both methyltransferase and demethylase gene expression increased (Figure 1F and S1), indicating that the Jarid1 family is responsible for the global downregulation of H3K4me3 upon differentiation. Jarid1b displayed the greatest increase among all four members of the Jarid1 family (Jarid1d is not shown due to a high Ct value). Western blotting confirmed the increase in Jarid1b (Figure 1E). In addition, we compared Jarid1 gene expression levels in human primary keratinocytes based on the GEO database (GSE21413) and found that Jarid1b expression was comparatively higher than that of the other two genes (Tables [Link], [Link], [Link]).

### 3.2 Jarid1b deregulates HaCaT cell differentiation

**Figure 2:**
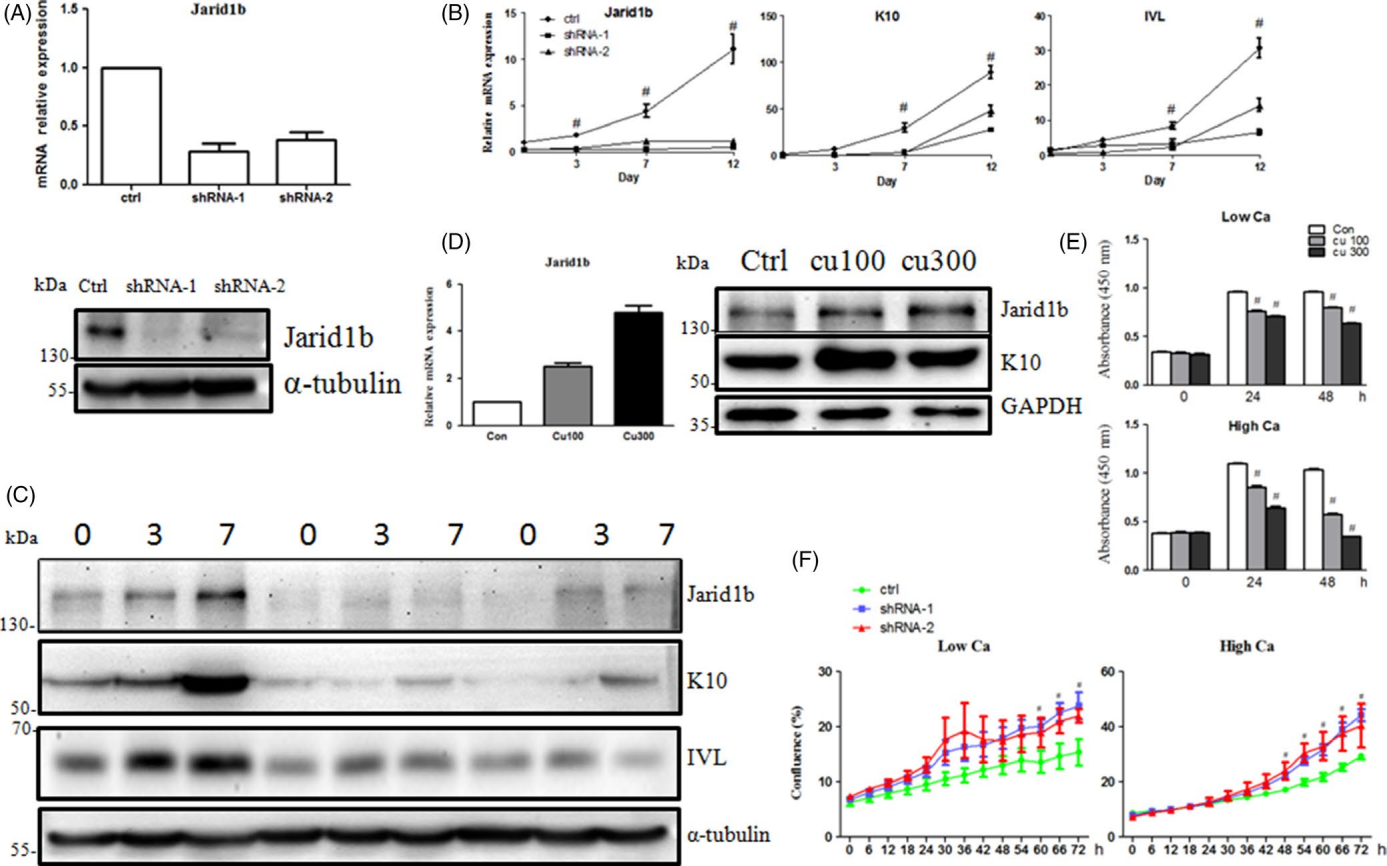
Jarid1b regulated HaCaT cell differentiation induced by high calcium. A, Expression of Jarid1b upon shRNA‐mediated knockdown at the mRNA and protein levels. B, RT‐qPCR and C, Western blot analysis of the expression of keratinocyte differentiation markers (K10 and IVL) upon Jarid1b silencing (#*P* < 0.01 shRNA group vs control at each time point, Student's *t* test). D, K10 protein expression upon cumate (100 and 300 μg/mL)‐induced Jarid1b overexpression in high‐calcium culture. E, HaCaT cell proliferation in low‐ and high‐calcium media upon Jarid1b overexpression or (F) knockdown (#*P* < 0.01 Jarid1b overexpression or knockdown group vs control at each time point, Student's *t* test)

Next, we silenced Jarid1b to explore its function in HaCaT cell differentiation. Jarid1b knockdown was confirmed at the mRNA and protein levels (Figure 2A). The mRNA and protein expression of K10 and IVL in Jarid1b knockdown cells was lower than that in the control cells at the indicated time points (Figure 2B,C), suggesting that Jarid1b silencing delayed HaCaT cell differentiation.

### 3.3 Overexpression of Jarid1b enhances HaCaT cell differentiation and inhibits proliferation

A stable, inducible Jarid1b‐overexpressing HaCaT cell line was produced by lentivirus infection followed by selection with 2 mg/mL puromycin to obtain a pure cell population. Jarid1b was overexpressed upon cumate treatment at the indicated concentration. K10 and IVL expression was upregulated with Jarid1b overexpression (Figure 2D and S2). In addition, the CCK8 assay showed that cell proliferation decreased upon cumate treatment compared with the control cells (Figure 2E), whereas knockdown of Jarid1b increased cell proliferation (Figure 2F), suggesting that the induction of Jarid1b may play a significant role in terminal differentiation.

### 3.4 Jarid1b silencing impairs terminal differentiation in reconstituted skin

**Figure 3:**
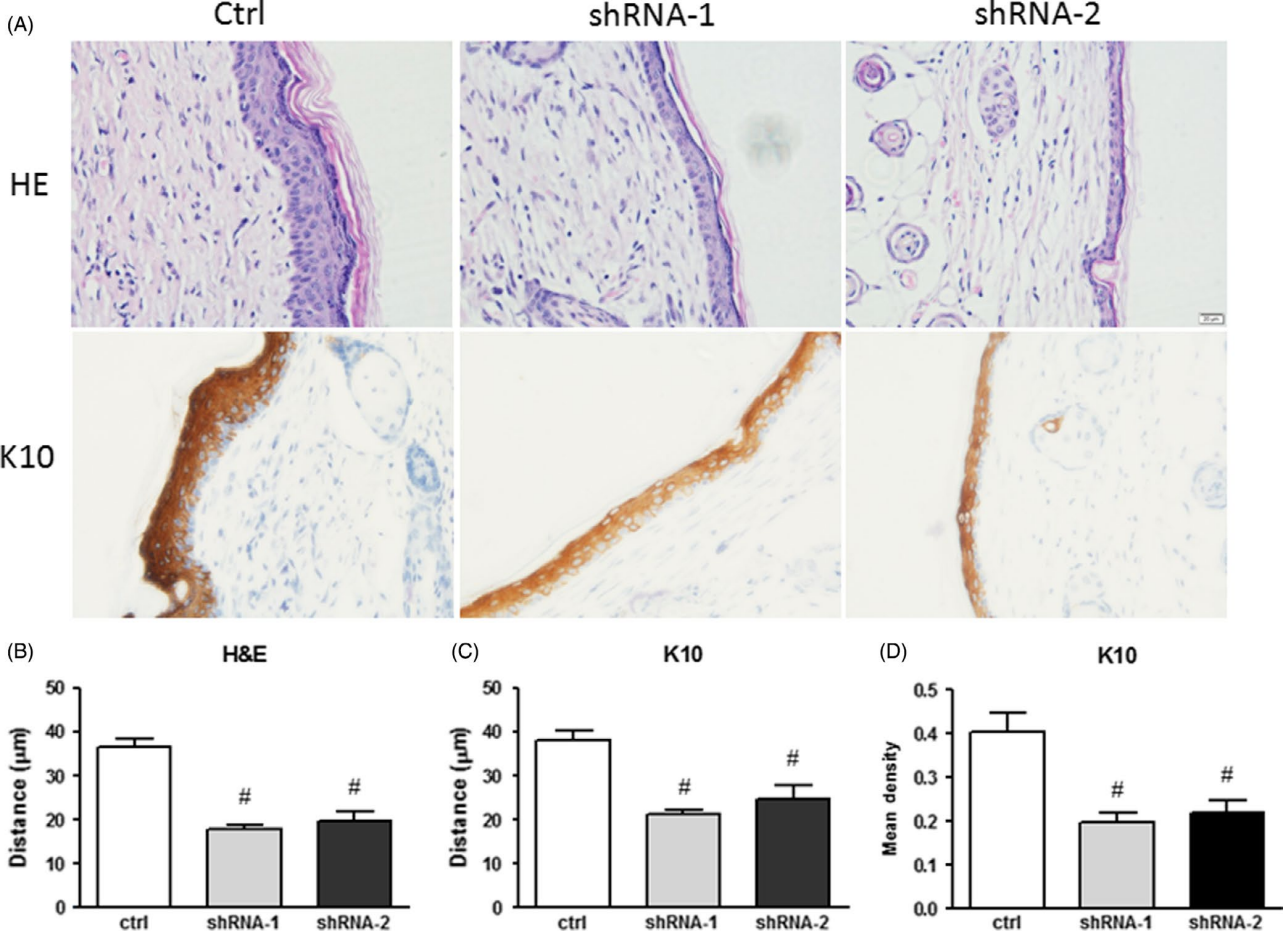
Depletion of Jarid1b impaired suprabasal layer formation. A, Representative images of H&E and K10 immunohistochemical staining in HaCaT reconstituted skin. B, Full‐thickness analysis of H&E staining in the epidermis. C, K10 staining thickness and (D) intensity analysis (#*P* < 0.01 shRNA group vs control at each time point, Student's *t* test)

To further understand the role of Jarid1b in vivo, HaCaT cells and mouse dermal fibroblasts were grafted onto nude mice.^19^ After 27 days, the reconstituted skin was analysed by histological and immunohistochemical assays. First, we confirmed that in the reconstituted skin, Jarid1b expression was depleted compared with the control (Figure S3). H&E staining showed that the control cell differentiated into well‐structured epithelia, whereas the Jarid1b‐silenced epidermal layers were thinner (Figure 3A,B). Moreover, staining for the differentiation marker K10 showed that the intensity was much lower and that the layer was thinner upon Jarid1b knockdown (Figure 3A,C,D), demonstrating substantial impairment of terminal differentiation. We also noted that all of the reconstituted epidermis consisted of one non‐K10‐stained basal cell layer (Figure 3A), though HaCaT cells retained K10 expression in the low‐calcium culture in vitro (Figure 1E), which showed that the transplantation assay was a reliable and sensitive model and that essential regulation by stoma interactions is needed for epidermis formation in vivo. This native epidermis‐resembling model strongly suggests a crucial role of Jarid1b in terminal differentiation.

### 3.5 Jarid1b controls MET gene expression

**Figure 4:**
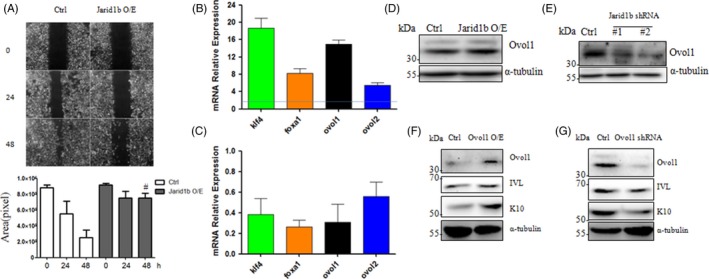
Jarid1b controlled MET gene expression. A, Representative images of wound healing scratches upon cumate‐induced Jarid1b overexpression at the indicated time points and quantification of the gap area (#*P* < 0.01 Jarid1b overexpression group vs control at each time point, Student's *t* test). B, Jaird1b overexpression and (C) knockdown regulated the expression of the MET gene (Klf4, Foxa1, Ovol1 and Ovol2). D, Jarid1b overexpression or (E) knockdown enhanced or inhibited Ovol1 protein expression, respectively. Western blot analysis of the expression of keratinocyte differentiation markers (K10 and IVL) upon Ovol1 (F) overexpression and (G) knockdown

To determine whether Jarid1b inhibits FaDu cell migration, a scratch wound healing assay was performed. Initially, both groups exhibited almost the same wound size at 0 hour. However, in the Jarid1b‐overexpressing group, the scratch wound area was still significantly larger than that in the control at 24 and 48 hours. Compared with the control, the cell migration rate was substantially decreased (*P* < 0.05) upon Jarid1b overexpression (Figure 4A). We speculated that Jarid1b could be involved in the epithelial‐mesenchymal transition (EMT) process, as Jarid1b contributes to cell migration and terminal differentiation. We found that the expression of mesenchymal‐epithelial transition (MET) genes, including Klf4, Foxa1, Ovol1 and Ovol2, was enhanced or suppressed upon Jarid1b overexpression or knockdown, respectively (Figure 4B,C). Western blot assays confirmed that Jarid1b overexpression resulted in elevated expression of Ovol1, whereas Jarid1b knockdown led to reduced Ovol1 expression (Figure 4D,E).

Recent studies have suggested that Ovol1 contributes to epidermal differentiation. Our results demonstrated that the expression of K10 and IVL was upregulated by Ovol1 overexpression (Figure 4F). Conversely, the expression of these genes was downregulated upon knockdown of Ovol1 (Figure 4G).

### 3.6 Ovol1 is controlled by the Jarid1b‐Ship1‐PI3K/AKT pathway

**Figure 5:**
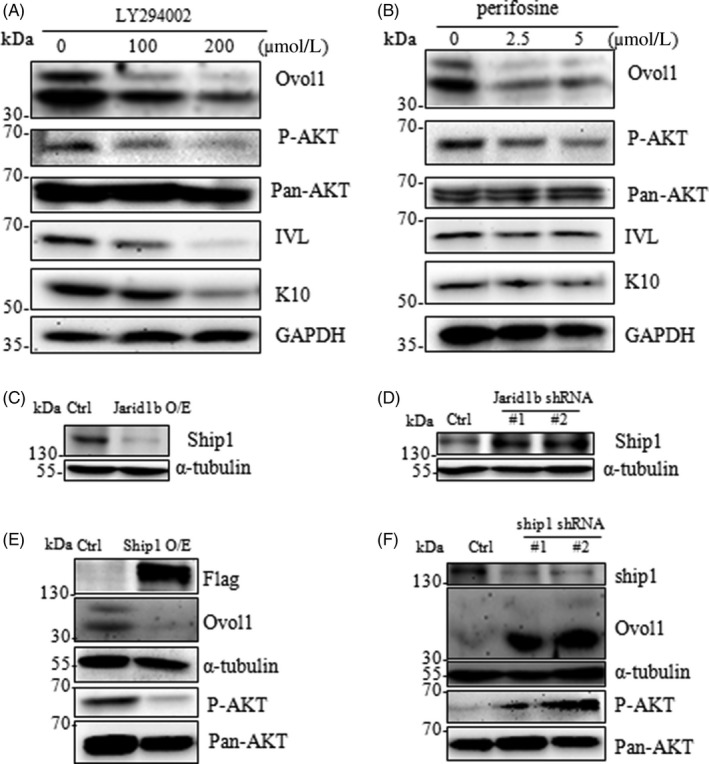
Ovol1 expression was regulated by the Jarid1b‐Ship1/PI3K/AKT pathway. A, The PI3K‐AKT inhibitors (A) LY294002 and (B) perifosine repressed the expression of Ovol1 and keratinocyte differentiation markers (K10 and IVL). C, Jarid1b overexpression and (D) knockdown promoted and repressed Ship1 expression, respectively. E, Overexpression of Ship1 inhibited AKT phosphorylation and Ovol1 expression. F, Silencing of Ship1 enhanced AKT phosphorylation and Ovol1 expression

Our previous study demonstrated that the PI3K‐AKT pathway promoted FaDu cell differentiation,^17^ which raised the possibility that Ovol1 could be involved in this process. Here, our results showed that Ovol1 expression was inhibited upon treatment with the PI3K inhibitor LY294002 and the AKT inhibitor perifosine, suggesting that PI3K‐AKT signalling plays an important role in the control of Ovol1 expression (Figure 5A,B). We also found that inhibition of PI3K/AKT signalling repressed the expression of the differentiation genes K10 and IVL (Figure 5A/B and S4a), which was consistent with our previous study in cancer cells. Jarid1b is a demethylase that functions as an eraser of H3K4me3, leading to transcription repression. Thus, there is less of a possibility that Jarid1b directly regulates Ovol1 transcription. We hypothesized that Jarid1b activated PI3K/AKT/Ovol1 by controlling suppressors of this pathway and that Ship1 could be one such potential candidate. Our results showed that Ship1 expression was repressed by Jarid1b overexpression and enhanced by Jarid1b depletion (Figure 5C,D). Furthermore, Ovol1 expression was decreased upon Ship1 overexpression and increased upon Ship1 knockdown (Figure 5E,F). We also overexpressed another important suppressor, Pten. Ectopic expression of Pten in HaCaT cells increased K10 expression (Figures S4B,C), indicating the specificity of Jarid1b‐Ship1 in the regulation of keratinocyte differentiation.

### 3.7 Jarid1b directly binds to the *SHIP1* gene promoter

**Figure 6:**
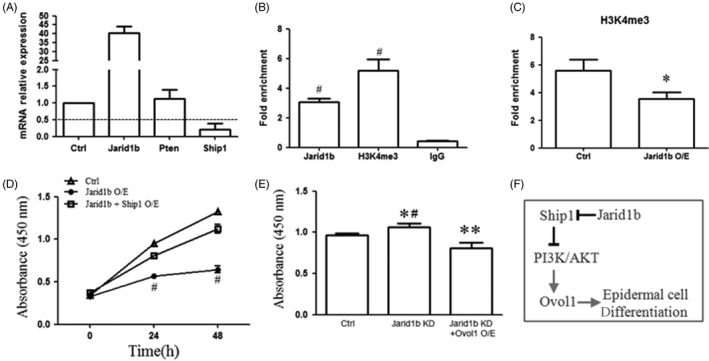
Jarid1b directly bound to the *SHIP1* gene promoter. A, mRNA expression of Ship1 and Pten upon Jarid1b overexpression. B, Jarid1b and H3K4me3 enrichment at the *SHIP1* gene promoter. C, H3K4me3 enrichment at the *SHIP1* gene promoter upon Jarid1b overexpression (*P < 0.05, normalized with IgG). Cell proliferation was measured by the CCK8 assay in (D) Jarid1b + control/Ship1‐Flag (#*P* < 0.01, Jarid1b O/E vs Jarid1b + Ship1 O/E) and (E) Jarid1b KD + control/Ovol1 O/E co‐transfected cells (*P < 0.05, Jarid1b KD vs control, #*P* < 0.01, Jarid1b KD vs Jarid1b KD + Ovol1 O/E, **P < 0.01, Jarid1b KD + Ovol1 O/E vs control, Student's *t* test). F, Experimental model explaining how Jarid1b regulates epidermal differentiation

Next, we examined whether another PI3K/AKT pathway suppressor, Pten, was regulated by Jarid1b. Unfortunately, there were no obvious changes in Pten upon Jarid1b overexpression (Figure 6A), suggesting that there is a specific mechanism by which Jarid1b regulates Ship1. We analysed the sequence of the *SHIP1* gene and found the consensus binding sequence (GCACA/C) of Jarid1b near its promoter.^22^ We designed primers containing the binding motif around the TSS gene to perform ChIP assays. Our results demonstrated that Jarid1b and H3K4me3 were enriched at the TSS of the *SHIP1* gene promoter (Figure 6B). Furthermore, overexpression of Jarid1b resulted in a reduction in H3K4me3 enrichment (Figure 6C). We next conducted a Ship1 rescue assay in Jarid1b‐overexpressing cells. The results showed that Ship1 overexpression could enhance cell proliferation, which was inhibited by Jarid1b overexpression (Figure 6D). In addition, Ovol1 overexpression attenuated the stimulation of cell proliferation by Jarid1b knockdown (Figure 6E). Taken together, our results demonstrated that Jarid1b directly binds the *SHIP1* gene by effectively activating the PI3K‐AKT‐Ovol1 pathway, resulting in keratinocyte differentiation.

## 4 DISCUSSION

Genome‐wide analyses have revealed that H3K4me3 is enriched at transcription start sites, which is regarded as an active histone marker. The global H3K4 methylation state is involved in the ESC fate decision during development.^23^, ^24^, ^25^ Global loss of H3K4me3 has been described during ESC differentiation.^6^ Our results showed that global H3K4me3 expression was decreased during differentiation in vitro and in vivo, which suggested that progenitor cell commitment is accompanied by H3K4me3 loss at the global level. Interestingly, LSC differentiation is also associated with stem maintenance gene repression via loss of H3K4me3, but not H3K79me2.^15^ Next, we explored the roles of H3K4‐specific demethylases and methyltransferases in the global loss of H3K4me3. Our results showed that the levels of both types of enzymes increased during calcium‐induced differentiation, indicating that only demethylases are responsible for the global loss of H3K4me3. The greatest increase was observed for Jarid1b among the four increased demethylases; Jarid1b has been reported to negatively regulate LSC stem potential and leukaemogenesis in murine and human MLL‐rearranged AML cells.^15^ A global reduction in H3K4me3 levels may be a common feature in stem cell specification, where stem gene silencing results from the removal of H3K4me3 by demethylases.

Next, our study demonstrated that the histone demethylase Jarid1b is a positive regulator of epidermal differentiation. This finding is consistent with the essential role of Jarid1b in ESC differentiation. Jarid1b is indispensable in the neural commitment of ESCs, though a minor effect on self‐renewal was observed.^11^ Knockdown of Jarid1b resulted in delayed ESC differentiation upon LIF depletion,^26^ which was also consistent with our results in vitro and in vivo. In addition, Jarid1b plays an important role in myogenic lineage specification from mesenchymal cells by regulating the enrichment of H3K4me3 at the Runx2 gene promoter.^10^ Here, we showed that Jarid1b controls Ship1 gene expression by directly binding to its promoter.

Our previous study demonstrated that Jarid1b overexpression is positively associated with hypopharyngeal squamous cell carcinoma (SCC) differentiation and that forced expression of Jarid1b leads to FaDu cell differentiation. Jarid1b has been reported to show elevated expression in various types of tumours, including breast,^27^ prostate^28^ and bladder^29^ tumours, and has been proposed as an oncogene. Controversially, Jarid1b has been reported as an epigenetic regulator with tumour‐suppressive potential in melanoma.^30^ A slow‐cycling melanoma cell subpopulation was shown to be characterized by high Jarid1b expression.^31^ In leukaemia, high expression of Jarid1b promotes cell differentiation, showing tumour‐suppressive potential rather than acting as an oncogene. Collectively, the available results indicate that the contribution of Jarid1b to tumours may be context‐dependent. Here, we demonstrated that Jarid1b contributed to epidermal cell differentiation. Considering these findings together with our previous results in FaDu cancer cells, we believe that Jarid1b may be a tumour suppressor rather than an oncogene, at least in squamous cell cancer carcinoma. For SCC differentiation, we usually have to use adjacent normal tissue as a control because the initial undifferentiated state has elapsed. Our study may provide a good example for characterizing gene function in SCC differentiation. From our results, we assume that SCC and epithelial cells may evolutionally share common mechanisms in promoting cell differentiation.

Jarid1b represents a barrier to ESC EMT and the reprogramming of differentiated cells by regulating mesenchymal master regulators,^32^ whereas in human lung and colon cancer cells, overexpression of Jarid1b promotes the EMT process via upregulating the expression of Zeb1 and Zeb2, which are targeted by microRNA‐200.^33^ Our results showed that Jarid1b overexpression impaired cell migration in a wound healing assay and promoted the expression of MET genes, among which Ovol1 has been proven to be a key molecule in epithelial differentiation. Ovol1 can repress Ovol2^34^ and its own transcription.^35^ Ovol1 knockout mice exhibit skin barrier impairment,^34^, ^36^ suggesting the important role of Ovol1 in epidermal differentiation. Filaggrin and involucrin levels are reduced upon Ovol1 knockout in vivo,^36^ which is consistent with our results regarding the regulation of Inv and K10. In skin melanoma, high expression of Ovol1 prevents tumour invasion and predicts a better prognosis than that associated with low expression.^37^ In cutaneous and oral SCC, the expression of Ovol1 markedly decreases in the invasive portion compared with the in situ state, implying a role of Ovol1 in potentiating differentiation.^38^, ^39^ Despite the importance in cell differentiation, the regulation of Ovol1 expression is largely unknown. NGN3 negatively regulates the transcription of Ovol1 in an E‐box‐dependent fashion in the rodent pancreas.^40^ In SCC cell lines, IRF6 binds to the Ovol1 gene promoter and positively regulates Ovol1 expression.^41^ Li et al^42^ showed that the mOvo1 promoter is activated by the LEF1/β‐catenin complex. Our previous research has shown that β‐catenin is regulated by the Jarid1b‐AKT pathway. Thus, we speculated that this pathway may also be involved in the regulation of Ovol1 expression. As expected, we found that Ovol1 expression was positively controlled by the Jarid1b‐PI3K‐AKT pathway. Interestingly, Jarid1b only controls the AKT pathway suppressor Ship1, and not Pten, by directly binding to its promoter, which suggests that there may be specific mechanisms to trigger distinct downstream biological effects.

Together, our studies clearly show the crucial role of Jarid1b in promoting epidermal cell differentiation (Figure 6F). As the observation of roles of Jarid1b in controlling cell differentiation and tumourigenesis remains controversial, a clear understanding of its roles in the regulation of differentiation in a tissue‐dependent system will be essential and beneficial for determining their functional involvement in SCC and, further, for developing proper therapeutic strategies in the future. In summary, our studies addressed a representative epithelial tissue, the skin, and demonstrated a clear role of Jarid1b‐mediated repression of Ship1 and activation of the AKT/Ovol1 pathway in enhancing the epithelial cell differentiation state.

## CONFLICT OF INTEREST

The authors declare no conflict of interest.

## AUTHOR CONTRIBUTIONS

ZJ, JY, YT, FT and AX designed the experiments and analysed the data; SX, NY, LZ, ZL, HY, LQ, ZS, CT and ZJ performed the research; and ZJ wrote the paper. All authors read and approved the final manuscript.

## References

[1] Zhang J , Bardot ES , Ezhkova E . Epigenetic regulation of skin: focus on the Polycomb complex. Cell Mol Life Sci. 2012;69(13):2161‐2172. 22314499 10.1007/s00018-012-0920-x PMC8284996

[2] Ezhkova E , Pasolli HA , Parker JS , et al. Ezh2 orchestrates gene expression for the stepwise differentiation of tissue‐specific stem cells. Cell. 2009;136(6):1122‐1135. 19303854 10.1016/j.cell.2008.12.043 PMC2716120

[3] Bardot ES , Valdes VJ , Zhang J , et al. Polycomb subunits Ezh1 and Ezh2 regulate the Merkel cell differentiation program in skin stem cells. EMBO J. 2013;32(14):1990‐2000. 23673358 10.1038/emboj.2013.110 PMC3715854

[4] Wurm S , Zhang J , Guinea‐Viniegra J , et al. Terminal epidermal differentiation is regulated by the interaction of Fra‐2/AP‐1 with Ezh2 and ERK1/2. Genes Dev. 2015;29(2):144‐156. 25547114 10.1101/gad.249748.114 PMC4298134

[5] Harikumar A , Meshorer E . Chromatin remodeling and bivalent histone modifications in embryonic stem cells. EMBO Rep. 2015;16(12):1609‐1619. 26553936 10.15252/embr.201541011 PMC4693513

[6] Efroni S , Duttagupta R , Cheng J , et al. Global transcription in pluripotent embryonic stem cells. Cell Stem Cell. 2008;2(5):437‐447. 18462694 10.1016/j.stem.2008.03.021 PMC2435228

[7] Mosammaparast N , Shi Y . Reversal of histone methylation: biochemical and molecular mechanisms of histone demethylases. Annu Rev Biochem. 2010;79:155‐179. 20373914 10.1146/annurev.biochem.78.070907.103946

[8] Yamane K , Tateishi K , Klose RJ , et al. PLU‐1 is an H3K4 demethylase involved in transcriptional repression and breast cancer cell proliferation. Mol Cell. 2007;25(6):801‐812. 17363312 10.1016/j.molcel.2007.03.001

[9] Pospisil V , Vargova K , Kokavec J , et al. Epigenetic silencing of the oncogenic miR‐17‐92 cluster during PU.1‐directed macrophage differentiation. EMBO J. 2011;30(21):4450‐4464. 21897363 10.1038/emboj.2011.317 PMC3230374

[10] Rojas A , Aguilar R , Henriquez B , et al. Epigenetic Control of the Bone‐master Runx2 Gene during Osteoblast‐lineage Commitment by the Histone Demethylase JARID1B/KDM5B. J Biol Chem. 2015;290(47):28329‐28342. 26453309 10.1074/jbc.M115.657825 PMC4653688

[11] Schmitz SU , Albert M , Malatesta M , et al. Jarid1b targets genes regulating development and is involved in neural differentiation. EMBO J. 2011;30(22):4586‐4600. 22020125 10.1038/emboj.2011.383 PMC3243600

[12] Cellot S , Hope KJ , Chagraoui J , et al. RNAi screen identifies Jarid1b as a major regulator of mouse HSC activity. Blood. 2013;122(9):1545‐1555. 23777767 10.1182/blood-2013-04-496281 PMC5289888

[13] Xu J , Kidder BL . KDM5B decommissions the H3K4 methylation landscape of self‐renewal genes during trophoblast stem cell differentiation. Biol Open. 2018;7(5):bio031245. 29748167 10.1242/bio.031245 PMC5992522

[14] Yamamoto S , Wu Z , Russnes H , et al. JARID1B is a luminal lineage‐driving oncogene in breast cancer. Cancer Cell. 2014;25(6):762‐777. 24937458 10.1016/j.ccr.2014.04.024 PMC4079039

[15] Wong S , Goode D , Iwasaki M , et al. The H3K4‐Methyl Epigenome regulates leukemia stem cell oncogenic potential. Cancer Cell. 2015;28(2):198‐209. 26190263 10.1016/j.ccell.2015.06.003 PMC4536132

[16] Nowak JA , Fuchs E . Isolation and culture of epithelial stem cells. Methods Mol Biol. 2009;482:215‐232. 19089359 10.1007/978-1-59745-060-7_14 PMC2760227

[17] Zhang J , An X , Han Y , et al. Overexpression of JARID1B promotes differentiation via SHIP1/AKT signaling in human hypopharyngeal squamous cell carcinoma. Cell Death Dis. 2016;7(9):e2358. 27584795 10.1038/cddis.2016.262 PMC5059865

[18] Lee TI , Johnstone SE , Young RA . Chromatin immunoprecipitation and microarray‐based analysis of protein location. Nat Protoc. 2006;1(2):729‐748. 17406303 10.1038/nprot.2006.98 PMC3004291

[19] Blanpain C , Lowry WE , Geoghegan A , Polak L , Fuchs E . Self‐renewal, multipotency, and the existence of two cell populations within an epithelial stem cell niche. Cell. 2004;118(5):635‐648. 15339667 10.1016/j.cell.2004.08.012

[20] Weinberg WC , Goodman LV , George C , et al. Reconstitution of hair follicle development in vivo: determination of follicle formation, hair growth, and hair quality by dermal cells. J Invest Dermatol. 1993;100(3):229‐236. 8440892 10.1111/1523-1747.ep12468971

[21] Colombo I , Sangiovanni E , Maggio R , et al. HaCaT cells as a reliable in vitro differentiation model to dissect the inflammatory/repair response of human keratinocytes. Mediators Inflamm. 2017;2017:7435621. 29391667 10.1155/2017/7435621 PMC5748104

[22] Scibetta AG , Santangelo S , Coleman J , et al. Functional analysis of the transcription repressor PLU‐1/JARID1B. Mol Cell Biol. 2007;27(20):7220‐7235. 17709396 10.1128/MCB.00274-07 PMC2168894

[23] Bernstein BE , Mikkelsen TS , Xie X , et al. A bivalent chromatin structure marks key developmental genes in embryonic stem cells. Cell. 2006;125(2):315‐326. 16630819 10.1016/j.cell.2006.02.041

[24] Mikkelsen TS , Ku M , Jaffe DB , et al. Genome‐wide maps of chromatin state in pluripotent and lineage‐committed cells. Nature. 2007;448(7153):553‐560. 17603471 10.1038/nature06008 PMC2921165

[25] Meissner A , Mikkelsen TS , Gu H , et al. Genome‐scale DNA methylation maps of pluripotent and differentiated cells. Nature. 2008;454(7205):766‐770. 18600261 10.1038/nature07107 PMC2896277

[26] Kidder BL , Hu G , Zhao K . KDM5B focuses H3K4 methylation near promoters and enhancers during embryonic stem cell self‐renewal and differentiation. Genome Biol. 2014;15(2):R32. 24495580 10.1186/gb-2014-15-2-r32 PMC4053761

[27] Lu PJ , Sundquist K , Baeckstrom D , et al. A novel gene (PLU‐1) containing highly conserved putative DNA/chromatin binding motifs is specifically up‐regulated in breast cancer. J Biol Chem. 1999;274(22):15633‐15645. 10336460 10.1074/jbc.274.22.15633

[28] Xiang Y , Zhu Z , Han G , et al. JARID1B is a histone H3 lysine 4 demethylase up‐regulated in prostate cancer. Proc Natl Acad Sci USA. 2007;104(49):19226‐19231. 18048344 10.1073/pnas.0700735104 PMC2148272

[29] Hayami S , Yoshimatsu M , Veerakumarasivam A , et al. Overexpression of the JmjC histone demethylase KDM5B in human carcinogenesis: involvement in the proliferation of cancer cells through the E2F/RB pathway. Mol Cancer. 2010;9:59. 20226085 10.1186/1476-4598-9-59 PMC2848192

[30] Roesch A , Mueller AM , Stempfl T , Moehle C , Landthaler M , Vogt T . RBP2‐H1/JARID1B is a transcriptional regulator with a tumor suppressive potential in melanoma cells. Int J Cancer. 2008;122(5):1047‐1057. 17973255 10.1002/ijc.23211

[31] Roesch A , Fukunaga‐Kalabis M , Schmidt EC , et al. A temporarily distinct subpopulation of slow‐cycling melanoma cells is required for continuous tumor growth. Cell. 2010;141(4):583‐594. 20478252 10.1016/j.cell.2010.04.020 PMC2882693

[32] Kidder BL , Hu G , Yu ZX , Liu C , Zhao K . Extended self‐renewal and accelerated reprogramming in the absence of Kdm5b. Mol Cell Biol. 2013;33(24):4793‐4810. 24100015 10.1128/MCB.00692-13 PMC3889548

[33] Enkhbaatar Z , Terashima M , Oktyabri D , et al. KDM5B histone demethylase controls epithelial‐mesenchymal transition of cancer cells by regulating the expression of the microRNA‐200 family. Cell Cycle. 2013;12(13):2100‐2112. 23759590 10.4161/cc.25142 PMC3737312

[34] Teng A , Nair M , Wells J , Segre JA , Dai X . Strain‐dependent perinatal lethality of Ovol1‐deficient mice and identification of Ovol2 as a downstream target of Ovol1 in skin epidermis. Biochem Biophys Acta. 2007;1772(1):89‐95. 17049212 10.1016/j.bbadis.2006.08.012 PMC1773004

[35] Nair M , Bilanchone V , Ortt K , Sinha S , Dai X . Ovol1 represses its own transcription by competing with transcription activator c‐Myb and by recruiting histone deacetylase activity. Nucleic Acids Res. 2007;35(5):1687‐1697. 17311813 10.1093/nar/gkl1141 PMC1865076

[36] Lee B , Villarreal‐Ponce A , Fallahi M , et al. Transcriptional mechanisms link epithelial plasticity to adhesion and differentiation of epidermal progenitor cells. Dev Cell. 2014;29(1):47‐58. 24735878 10.1016/j.devcel.2014.03.005 PMC4153751

[37] Wouters J , Vizoso M , Martinez‐Cardus A , et al. Comprehensive DNA methylation study identifies novel progression‐related and prognostic markers for cutaneous melanoma. BMC Med. 2017;15(1):101. 28578692 10.1186/s12916-017-0851-3 PMC5458482

[38] Ito T , Tsuji G , Ohno F , Nakahara T , Uchi H , Furue M . Potential role of the OVOL1‐OVOL2 axis and c‐Myc in the progression of cutaneous squamous cell carcinoma. Mod Pathol. 2017;30(7):919‐927. 28339425 10.1038/modpathol.2016.169

[39] Jensen DH , Dabelsteen E , Specht L , et al. Molecular profiling of tumour budding implicates TGFbeta‐mediated epithelial‐mesenchymal transition as a therapeutic target in oral squamous cell carcinoma. J Pathol. 2015;236(4):505‐516. 25925492 10.1002/path.4550

[40] Vetere A , Li W‐C , Paroni F , et al. OVO homologue‐like 1 (Ovol1) transcription factor: a novel target of neurogenin‐3 in rodent pancreas. Diabetologia. 2010;53(1):115‐122. 19882138 10.1007/s00125-009-1567-5 PMC3066144

[41] Botti E , Spallone G , Moretti F , et al. Developmental factor IRF6 exhibits tumor suppressor activity in squamous cell carcinomas. Proc Natl Acad Sci USA. 2011;108(33):13710‐13715. 21807998 10.1073/pnas.1110931108 PMC3158164

[42] Li B , Mackay DR , Dai Q , et al. The LEF1/beta ‐catenin complex activates movo1, a mouse homolog of Drosophila ovo required for epidermal appendage differentiation. Proc Natl Acad Sci USA. 2002;99(9):6064‐6069. 11983900 10.1073/pnas.092137099 PMC122902

