# Supplementary figures and images for: Jarid1b promotes epidermal differentiation by mediating the repression of Ship1 and activation of the AKT/Ovol1 pathway
Source: Cell Prolif. 2019 May 31;52(5):e12638. doi: 10.1111/cpr.12638 (PMC6797505; doi:10.1111/cpr.12638)

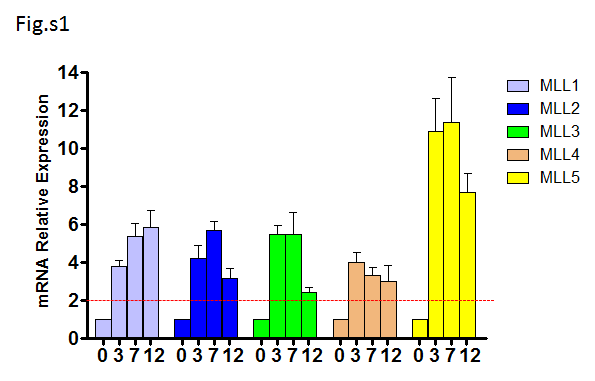

Supplement: Supplementary file 1 [file CPR-52-e12638-s001.tif]

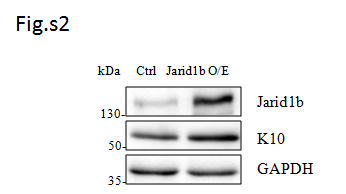

Supplement: Supplementary file 2 [file CPR-52-e12638-s002.tif]

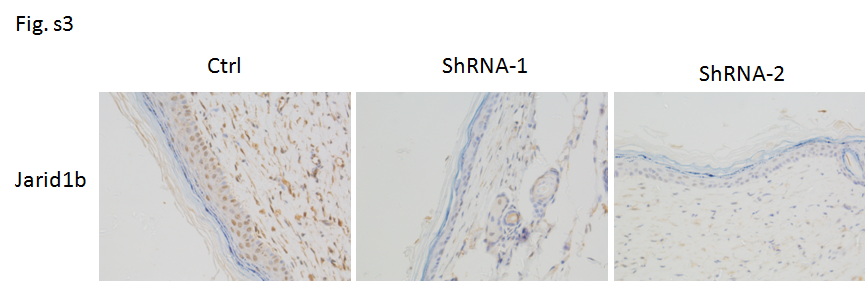

Supplement: Supplementary file 3 [file CPR-52-e12638-s003.tif]

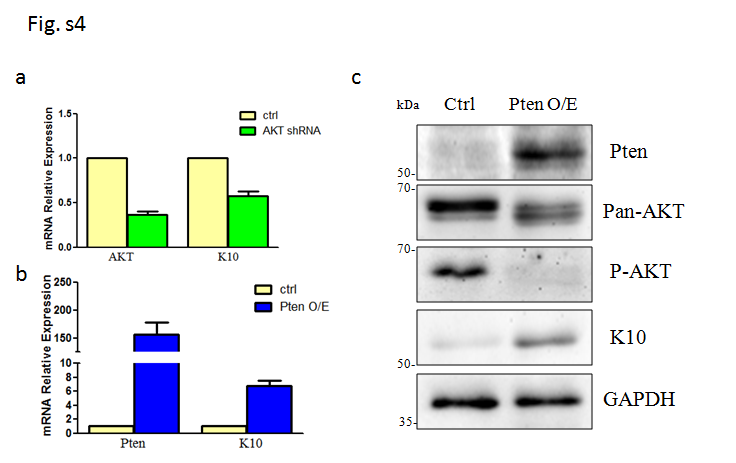

Supplement: Supplementary file 4 [file CPR-52-e12638-s004.tif]
