# Supplementary material for: Jarid1b promotes epidermal differentiation by mediating the repression of Ship1 and activation of the AKT/Ovol1 pathway
Source: Cell Prolif. 2019 May 31;52(5):e12638. doi: 10.1111/cpr.12638 (PMC6797505; doi:10.1111/cpr.12638)
Supplement: Supplementary file 8 [file CPR-52-e12638-s008.docx]

1. Supplementary Table legend for “Comparison of Jarid1 family member from GSE21413”

Jarid1 gene expression analysis of primary human keratinocytes with low or high calcium culture from GEO dataset (GSE21413).

1. Supplementary Table legend for “PrimerList”

The list of primers used for qPCR or ChIP-PCR.

1. Supplementary Table legend for “AntibodyList”

The list of antibodies used for Western Blot or IHC.
